# Vulnerability of smallholder sorghum farmers to climate variability in a heterogeneous landscape of south-western Uganda

**DOI:** 10.4102/jamba.v12i1.849

**Published:** 2020-04-30

**Authors:** Frank Mugagga, Noeline Nakanjakko, Bob Nakileza, Denis Nseka

**Affiliations:** 1 Department of Geography, Geo-Informatics and Climatic Sciences, College of Agricultural and Environmental Sciences, Makerere University, Kampala, Uganda; 2 Department of Environmental Management, College of Agricultural and Environmental Sciences, Makerere University, Kampala, Uganda

**Keywords:** Climate variability, altitudinal variations, Kigezi highlands, targeted interventions, vulnerability index

## Abstract

Smallholder farmers in sub-Saharan Africa are at a greater risk to the impacts of climate variability. We therefore sought to assess vulnerability of smallholder sorghum farmers to climate variability in Kigezi highlands of south-western Uganda. A vulnerability index that integrates selected socio-economic and biophysical variables was obtained through key informant interviews and household surveys, from 230 conveniently sampled sorghum farming households within three sub-counties differentiated by altitude. Rainfall data were obtained from Uganda National Meteorological Authority. Quantitative data were analysed using Statistical Package for Social Sciences (version 23) and STATA software to generate inferential and descriptive statistics, notably frequencies, percentages and chi-square tests, to establish relationship between variables. Content analysis was used to generate themes emerging from the qualitative data. The overall vulnerability index results indicate Kashambya as the most vulnerable (6.9), followed by Bubare (1.8), while Kamwezi was the least vulnerable (–0.2). This study recommends targeted extension services such as access to customised weather information and better agronomic practices to reduce smallholder sorghum farmers’ vulnerability.

## Introduction

Climate variability is probably the most complex and challenging environmental problem facing the world today (IPCC 2014; Zizinga et al. 2015). The effects of climate variability are predicted to be more felt by the largely vulnerable smallholder farmers. Smallholder farmers in developing countries are highly vulnerable to variations and changes in climate (Bennett & Vanwey 2015; IPCC 2014) because of poverty and high marginalisation (Bennett & Vanwey 2015). Vulnerability is the degree to which a system is susceptible to, or unable to cope with, the adverse effects of climate change including climate variability and its extremes (IPCC 2014). It is a function of the character, magnitude and rate of climate variation to which a system is exposed, its sensitivity and its adaptive capacity. The determinants of adaptive capacity are directly correlated with measures of economic development (GDP per *capita*), and Africa has been noted to be already under pressure from climate stresses because of, among other things, lack of institutional capacity usually interpreted as lack of governance capacity (IPCC 2014). Effects of climate variability are predicted to be more felt in the mountainous regions compared to lowlands because of decreasing lapse rate (Viviroli et al. 2011). This is because mountainous areas are agriculturally rich and contain some of the world’s highest rural population densities. For instance, the Virunga volcano region of Rwanda recorded a population density of 400 people per km^2^, and Mount Elgon slopes had 700 people per km^2^ (UBOS 2016). The population density of Kigezi highlands was 320 people per km^2^ as per UBOS (2016), thus putting mountain and highland resources under stress and people at risk to climate variability extremes such as long dry spells.

Among the key crops grown in the highlands of sub-Saharan Africa is sorghum, which is also the world’s fifth most important cereal after wheat, maize, rice and barley (Ogeto et al. 2013). It is Africa’s second most important cereal crop after maize (Macauley 2015). Its production has, however, not kept pace with the increasing demand (Macauley 2015). Statistically, sorghum production trends have been decreasing since 2012. Gebrekiros, Araya and Yemane (2016) reported a decrease in sorghum yields by 24% in Tigray, Ethiopia. The consequences of this decrease are huge drops in household welfare, especially significant reductions in household consumption and asset depletion (Macours 2013). It can also hamper households’ nutrition, productivity and upward mobility, with intergenerational consequences.

The south-western highlands where the bulk of sorghum is grown in the country greatly vary in altitude from low, moderate to higher elevation and this dictates the nature of likely hazards that may hit smallholder farmers such as floods, dry spells and strong winds. The objective of this study was to assess how vulnerable smallholder sorghum farmers in Kigezi highlands are to climate variability. It was hypothesised that the vulnerability of smallholder sorghum farmers significantly varies across the altitudinal zones.

## Materials and methods

### Study area

**FIGURE 1:**
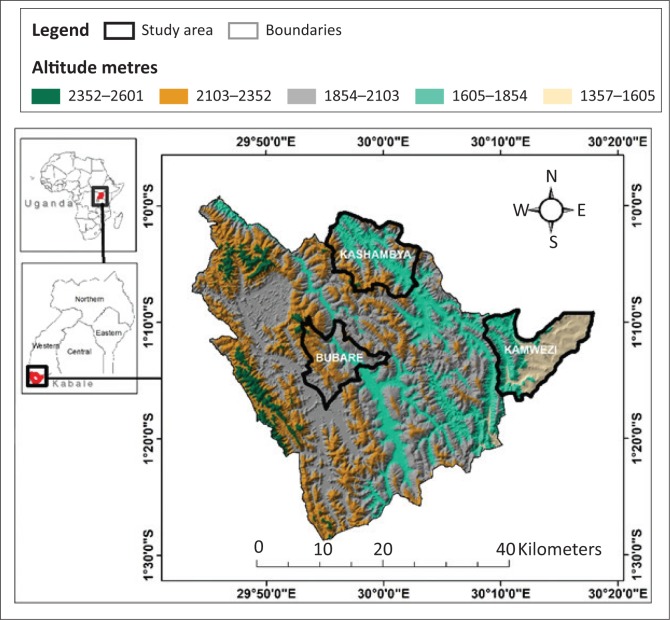
Location of the study area.

This study was carried out in the Kigezi highlands in south-western Uganda, located between longitude 29^o^ 45’ and 30^o^ 15’ east and 1^o^ 00’ and latitude 1^o^ 29’ south (Figure 1).

This study concentrated on three altitude zones, namely Bubare, Kashambya and Kamwezi, lying within altitudes of 1800 m – 2080 m, 1800 m – 1887 m and 1500 m – 1693 m above the sea level. The area’s topography greatly varies with flatlands, valleys, hills and highlands. The area receives more than 1200 mm of annual rainfall (Twagiramaria & Tolo 2016) and thus has a montane climate.

Temperatures average about 18 °C during the day and fall to about 10°C at night. Relative humidity is between 90% and 100% in the mornings and decreases to between 42% and 75% in the afternoon, throughout the year (KDLG 2015). It has two main rainy seasons of March to May as the heavy rains and September to November as light rains with intervals of some dry spells.

In the Kabale highlands where this study concentrated, the rocks are of Ankole-Karegwean nature composed of phyllites, shales, mudstones, quartzites and metamorphic-like schists, while the soils are dark red and brown sandy loam and sandy clay loam (Bagoora 1988). Kigezi highland area records one of the highest population densities (UBOS 2016), with highly fragmented pieces of land (Bagoora 1988; Twagiramaria & Tolo 2016). Sorghum was investigated because it is a traditional cash and food crop in the area, evidently challenged by the effects of climate change (Akatwijuka, Rubaihayo & Odong 2016).

## Study procedure

A description of the indices and procedures is elaborated below. Exposure in this context was defined as the nature and degree of contact of smallholder sorghum farmers to long dry spells (IPCC 2014). Exposure was reflected in magnitude and rate of the climate variability extreme that the farmers had experienced. Climate variability impacts are predicted to have far-reaching impacts in mountainous areas because of decreasing lapse rate (Viviroli et al. 2011). The altitudinal variation, further, has a bearing on intensity and distribution of rainfall in that areas on higher altitude are likely to receive more intense rains and consequential erosion of the landscape, while the valley bottoms may face problems of flooding.

Farmers’ perceptions on rainfall and temperature for the last 27 years triangulated with Focused Group Discussions (FGDs) conducted with elders were used to deduce the exposure of smallholder sorghum farmers to climate variability. This study investigated farmers’ perceptions of climate variability impacts for a period of 27 years as far as smallholder farmers could recall. Farmers could recall long dry spells effects from 1992. However, it was only possible to get data from Uganda National Metrological Authority from 1992 to 2014.

Data were collected on eight indicators that included type of shock (long dry spells), time of shock, annual frequency of occurrence of these climatic shocks, observations made in rainy seasons in the last 10–15 years, observations made in temperatures in the last 10–15 years, when the last major long dry spell was experienced, cause of this climate variability or seasonal variability and trend of rainfall over the last 15 years. However, given the subjectivity of farmers’ perceptions, data from UNMA were relied on. The exposure index was generated in STATA using principal component analysis (PCA) following the procedure described in the methodology.

Sensitivity is defined as the degree to which a system is affected either beneficially or adversely by climate-related stimuli (IPCC 2014). The system’s sensitivity is determined by internal conditions which are mainly the socio-economic variables and some external biophysical elements (Füssel & Klein 2006). There are particular elements of the social–ecological system that are most sensitive to climate hazards and are considered most important. These depend on spatial development patterns, as well as cultural preferences, attitudes toward nature/biodiversity and reliance on climate-sensitive resources or services among other factors (Adger 2006). Data were collected on 11 variables, namely type of land holding, the number of plots owned, security of land holding, varieties of sorghum grown, size of the land allocated to sorghum growing in terms of plots, distance from home to the garden, amount of sorghum harvested in the base year, average income from sorghum, nature of house roof, nature of house wall and the number of children. Yield data of sorghum could not be captured because of the absence of records by smallholder sorghum farmers and at the Kabale District Production Office.

Adaptive capacity as defined by the IPCC (2014) refers to the potential, capability or ability of a system to cope with climate variability effects or impacts (Abdul-Razak & Kruse 2017). Numerous indicators have been developed, including education, income, health as well as access to financial, technological and institutional resources. More than 21 variables (Table 5) were analysed to establish how they shape adaptive capacity of smallholder sorghum farmers.

Firstly, normalisation of the proxy variables (e.g. education level, distance from home to garden, the number of children) was done based on the United Nations Development Programme (UNDP) Human Development Index (HDI) framework to ensure that values are within a comparable range because indicators have different scales and units. Normalisation ensures that values fall between 0 and 1.

The formula below was applied to normalise the indicators (UNDP 2016):

$$X_{\text{ij}}=(X_{\text{ij}}-X_{\text{i}})/\sigma_{\text{ij}}$$ [Eqn 1]

where *X*_ij_ represents the observed value of the variable (indicators), *X*_i_ represents the mean value of the indicator and σij represents the standard deviation for each indicator.

After normalising the data, weights were assigned using PCA. Principal component analysis has been used by different scholars; however, this study adapted the procedure used by Glwadys et al. (2010). Principal component analysis is a dimension reduction tool that can be used to reduce a large set of variables to a small set that still contains most of the information in the large set. The factor loadings or scores from the first principal (PC), which accounts for the largest variation in the data set, are considered as weights for the indicators. These factor loadings measure the extent to which each indicator contributes to the resulting principal component and provide information to the diversity of vulnerability. The first principal component is used to assign weights and construct an overall index (Equation 2):

V j=∑[bi(a ji−xi)] sii=1 i=1…k; j=1 … j [Eqn 2]

where *v* is the vulnerability index, *b* is the weight from PCA, *i* is the indicator value, *x* is the mean indicator value, *s* is the standard deviation of the indicators, *i* is the indicator and *j* is the specific altitudinal zone.

Using the IPCC (2014) definition of vulnerability, the overall vulnerability index was computed as:

$$V_{\text{shs}}=f(E_{\text{shs}}+S_{\text{shs}}-{\text{AC}}_{\text{shs}}),$$ [Eqn 3]

where *V*_shs_ is the vulnerability of smallholder sorghum farmers, *E*_shs_ is the exposure of smallholder sorghum farmers, *S*_shs_ is the sensitivity of smallholder sorghum farmers and AC_shs_ is the adaptive capacity of smallholder sorghum farmers.

### Ethical consideration

The study was approved by Makerere University’s Research Ethics Review Board (ERB). Respondents’ prior consent was sought and their privacy was ensured by keeping responses anonymous.

## Results

### Exposure to climate variability hazards

From the analysis, type of shock (particularly dry spells), time of shock, frequency of climatic shocks, rainfall and temperature trends were extracted as the most notable exposure indicators as presented in Table 1.

**TABLE 1:** Factor loadings for exposure of small-scale sorghum farmers to climate variability in the highlands of south-western Uganda.

Exposure indicator	Component 1	Mean	SD
Type of shock (long dry spells)	0.290	7.66	1.489
Time of shock	−0.033	2016.70	2.083
How often do these climatic shocks occur in a year?	0.426	1.35	0.479
Observations made in rain seasons in the last 10–15 years	0.334	2.04	1.548
Observations made in temperatures in the last 10–15 years	0.541	2.55	0.811
When was the last major long dry spells experienced?	−0.044	2015.81	4.315
Trend of rainfall over the last 10–15 years	0.938	1.75	0.483

SD, standard deviation.

Based on the comparative exposure index (Table 2), Bubare was the most exposed to climate variability hazards (2.4), followed by Kashambya (2.3) and Kamwezi (0.7). The mean difference in the level of exposure of the three altitudinal zones was significant (*p* < 0.05).

**TABLE 2:** Comparative exposure index of the altitudinal zones.

Altitudinal Zone	*N*	Mean	SD	*F*	*p*
Bubare	85	2.4	5.5	-	-
Kashambya	70	2.3	4.4	3.67	0.027
Kamwezi	75	0.7	2.2	-	-
Total	230	1.8	4.4	-	-

SD, standard deviation.

### Sensitivity to climate variability hazards

Out of the 11 variables investigated, the number of plots owned, distance of gardens from home, amount of sorghum harvested, nature of house wall and source of water were extracted by PCA as shown in Table 3.

**TABLE 3:** Factor loadings for sensitivity.

Sensitivity indicator	Component1	Mean	SD
Type of land holding	0.017	2.23	0.549
No. of plots owned	0.765	4.26	3.353
Do you feel secure about your land holding?	0.038	1.08	0.269
Species of sorghum grown	0.137	4.75	3.568
Size of the land allocated to sorghum growing in terms of plots	−0.169	1.5882	1.13424
Distance of garden from home (km)	0.766	1.8621	2.59441
Kilograms harvested from sorghum	0.739	300.9417	226.29908
Average income from sorghum	−0.313	1.64	0.772
Nature of house roof	−0.384	1.07	0.255
Type of toilets	−0.309	1.96	0.258
Nature of house wall	0.162	1.89	0.312
Number of children	−0.046	3.69	1.664
Source of water	0.781	1.82	0.896

SD, standard deviation.

Generally, the major type of landholding in the study area is customary. Most houses in the area have temporary floors and walls. A negligible number of households have cemented floors with permanent walls. Water sources varied within the study area, with more spring water in Kashambya, while Kamwezi and Bubare had both gravitational and spring water. The number of plots allocated to sorghum growing varied from one household to another, depending on the number of plots owned.

Sensitivity varied in the three altitudinal zones (Table 4), with results indicating Kashambya as the most sensitive (2.9), followed by Bubare (1.9) and lastly Kamwezi (0.4). The mean difference in sensitivity levels was significant at *p* = 0.04 < 0.05.

**TABLE 4:** Comparative sensitivity index by altitudinal zone.

Altitudinal zone	*N*	Mean	SD	*F*	*p*
Bubare	85	1.9	6.1	-	-
Kashambya	70	2.9	8.5	3.16	0.04
Kamwezi	75	0.4	2.2	-	-
**Total**	**230**	**1.7**	**6.2**	**-**	**-**

SD, standard deviation.

### Adaptive capacity of households to climate variability hazards

Out of the 21 variables assessed, 9 (42%) were extracted by PCA, namely, education level, household possessions, the number of animals, receipt of farmer-to-farmer extension services, change of farming practices in the last 15 years, engagement in group activities (including conservation activities), government strategies put in the area to address climate hazards and annual income variables (Table 5).

**TABLE 5:** Factor loadings for adaptive capacity.

Adaptive capacity indicator	Component1	Mean	SD
Age of respondent	0.076	3.24	1.530
Sex	−0.539	1.65	0.479
Relation	−0.429	1.65	0.884
Marital status	−0.303	1.56	1.161
Education level	0.273	1.98	0.733
Primary activity	0.014	1.21	0.816
Household possessions	0.431	0.94	1.100
No. of animals	0.392	4.25	6.321
Non-farm activities	−0.193	4.29	2.430
Type of transport used when transporting the produce	−0.355	2.30	0.986
Type of extension services got	−0.099	2.97	0.236
Do you have access to information on climate or seasonal variability?	0.094	0.39	0.489
Do you receive farmer to farmer extension services?	0.150	0.13	0.333
Sources of credit	−0.028	2.89	1.430
Have you ever changed your farming ways in the last 15 years?	0.402	0.27	0.445
Household member belonging to a group or groups of people doing conservation activities	0.642	0.77	0.422
Any group activities	0.483	0.83	0.372
Do you have others related to soil and water management?	−0.196	0.94	0.237
Benefit of belonging to the identified groups	−0.102	0.91	0.538
Strategies the government put in the area to address climate hazards	0.427	2.89	0.727
Social, development or self-help group membership	−0.343	3.70	2.324
Aggregated annual income	0.329	400 304.35	562 780.367

SD, standard deviation.

The adaptive capacity index presents Bubare with highest (2.0) adaptive capacity, followed by Kamwezi (0.9) and Kashambya (−1.1). There was no significant difference (*p* > 0.05) in the level of adaptive capacity across the altitudinal zones.

## Discussion

### Vulnerability as a function of altitude

Kamwezi, which is on lower altitude, experiences two dry seasons, while Kashambya and Bubare, which are on relatively higher altitude, are affected by one longer dry spell. This resonates with findings from other studies (e.g. McGuire 2000). The climatic conditions in Bubare and Kashambya could be modified by the mountainous landscape towards the south of Uganda, while the dry conditions of Kamwezi could be attributed to the cattle corridor bordering the north-east.

### Women’s tenure insecurity as precursor for increased vulnerability

The dominance of customary tenure across the study area poses problems of tenure insecurity, given less interest to invest in soil conservation initiatives (Mugagga 2017). The issues of tenure insecurity and women involvement are very relevant to understanding determinants of vulnerability. For instance, the motivation to invest in long-term conservation practices (which are also key to addressing climate change vulnerability) is often a subject of tenure security whereby farmers whose land is secured will be motivated to invest in long-term conservation as compared to those who are less secure (Mugagga 2017; Mugagga & Buyinza 2013). Furthermore, it is a known reality in Kigezi highlands that women access the land, more so for sorghum growing; however, they have less control over the land, given the cultural dispositions that favour men. Such a situation puts women in a more vulnerable situation, with precarious implications for sorghum production.

### Livelihood diversification as an adaptive strategy

As an adaptive strategy, smallholder sorghum farmers keep a myriad of livestock units, including cattle, goats, sheep, pigs and chicken, as sources of cash income and security (Table 5). Ogallo (2014) also noted in his study on household vulnerability and adaptive capacity to impacts of climate change and variability in Soroti District, Eastern Uganda, that farmer households relied on sale of livestock as an adaptive strategy.

### The potential of saving groups to reduce vulnerability

Availability of and membership to support groups such as saving cooperatives have been noted to spur response actions in the face of climate variability (Mugagga 2017). In the study area, households that had one or more of its members, particularly women belonging to a savings group, were evidently less vulnerable. For instance, through these groups, families were able to secure water tanks that were used to harvest water such that during a dry spell when streams get dried up, the households have had water for domestic use (Table 5). This resonates with studies in Kenya by Mburu (2016), which noted that many farmers use group savings to pull resources and buy water tanks for each member in return. Findings by Wanjiru (2016) on Mt. Kenya region also revealed that many farmers used group saving to pull resources and buy water tanks for each member in turn, thereby reducing vulnerability in case of a dry spell.

### Access to weather information and extension services

Much as this study found access to weather information insignificant, evidence elsewhere points to the importance of such information in enhancing the adaptive capacity of smallholder farmers in making farm management decisions such as when to plant, what to plant, where and how to plant (IPCC 2014), hence reducing on vulnerability levels. For sorghum in particular, weather information is needed by farmers to be able to plant the crop in the same period for controlling the effects of birds.

Extension services are a key factor in strengthening adaptive capacity through knowledge building. According to Mkisi and Peters (2014), extension services assist farmers in capacity development through adult and non-formal education. They facilitate and implement new programmes, act as feedback to the government and other interested agencies, enhance farmers’ knowledge and skills in climate change and adaptation-related practices and technologies. Study findings from a key informant at the Kacwenkano Zonal Agricultural Institute and the District Agricultural Officers indicated that their services had not reached out to smallholder sorghum farmers but research in new sorghum hybrids was underway and would soon reach out to the farmers. There is also a new sorghum agronomic practice of line planting considered to be more economical although farmers claimed it was more tedious and time wasting compared to the traditional broadcasting system. However, farmers from Kamwezi reported that they had not received any extension services, while those from Kashambya altitudinal zone reported to have got information on weather forecast from their sub-county chairperson.

## Conclusion and recommendations

Smallholder sorghum farmers in Kigezi highlands are highly vulnerable to climate variability hazards, though to differing degrees with respect to altitude. Kashambya, in particular, is presented as the most sensitive and most vulnerable because of low adaptive capacity compared to other areas. The vulnerability of these areas could be as a result of limited access to weather information and extension services. This study therefore recommends targeted extension services such as access to customised weather information and better agronomic practices to reduce smallholder sorghum farmers’ vulnerability.
